# Psychometric Properties of the Brazilian Version of Environmental Protectors Against Hospital Work Stress

**DOI:** 10.3390/healthcare13131618

**Published:** 2025-07-07

**Authors:** Silmara Meneguin, Paula Astudillo-Díaz, José Fausto de Morais, Camila Fernandes Pollo, Juliana Pierami de Freitas, Cesar de Oliveira, Aniele Fernanda Deplácido De Léo

**Affiliations:** 1 Department of Nursing, Botucatu Medical School, São Paulo State University (UNESP), Botucatu 18618-687, SP, Brazil; camilapollo@hotmail.com (C.F.P.); juliana.pierami@unesp.br (J.P.d.F.); anieledeleo@gmail.com (A.F.D.D.L.); 2 Department of Nursing, Universidad de la Frontera, Temuco 4811230, Chile; paula.astudillo@ufrontera.cl; 3 Institute of Mathematics and Statistics, Federal University of Uberlândia, Uberlândia 38405-320, MG, Brazil; jfmorais.ufu@hotmail.com; 4 Department of Epidemiology & Public Health, University College London, London WC1E 6BT, UK; c.oliveira@ucl.ac.uk

**Keywords:** occupational stress, psychometrics, health personnel, occupational health, hospitals

## Abstract

**Objective**: To analyse the psychometric properties of the Brazilian version of the Environmental Protectors Against Hospital Work Stress (ENPROS). **Methods**: A cross-sectional methodological study was conducted in two public hospitals in São Paulo state, Brazil, involving 431 doctors and nursing staff. Exploratory and confirmatory factor analyses were performed to assess the construct validity of the instrument. To evaluate internal consistency and measurement stability, Cronbach’s alpha and the intraclass correlation coefficient (ICC) were used. Convergent validity was tested by comparison with the Job Stress Scale. **Results**: Exploratory factor analysis indicated a structure comprising four factors, with satisfactory factor loadings and commonalities ranging from 0.26 to 0.75. Confirmatory factor analysis suggested a model with good fit (CFI = 0.988; TLI = 0.988; RMSEA = 0.064; SRMR = 0.067). Cronbach’s alpha was 0.95, and the intraclass correlation coefficient (ICC) was 0.45. Convergent validity showed significant correlations between the ENPROS domains and the Job Stress Scale factors, particularly in the social support domain. **Conclusions**: The Brazilian Portuguese version of the Environmental Protectors Against Hospital Work Stress scale has a four-dimensional structure, adequate reliability, and a good fit to the proposed factorial model.

## 1. Introduction

According to the US National Institute for Occupational Safety and Health, occupational stress involves a variety of negative physical and emotional responses that occur when job demands surpass the capabilities, resources, and needs of workers [1]. This form of stress is recognised as a major occupational health concern affecting thousands of workers worldwide [2]. 

The hospital workplace is a significant source of stress due to low wages, complex human relationships, a shortage of materials, an inadequate number of staff [3], long working hours, and intense activities, which can compromise both staff health and the quality of care provided to patients [4]. Studies on occupational stress suggest its negative impact on patient safety because of the increased risk of adverse events linked to falls, medication errors, and infection. Occupational stress can also affect the workforce by disrupting employment ties or reducing performance during working hours [5,6]. Prolonged exposure to stress can result in impaired work performance and contribute to the development of burnout syndrome, substance abuse, and suicide among workers [7,8]. 

Evidence also indicates that occupational stress negatively affects both the technical and non-technical performance of hospital staff, especially during critical situations like surgery, thereby directly influencing clinical outcomes. Furthermore, the decline in decision-making, communication, and teamwork can endanger patient safety, with up to one-third of communication errors linked to failures that could cause harm [9,10].

A recent study has shown that occupational stress among health professionals remains affected by occupational policies, local culture, and ambience, mainly characterised by workload, precarious working conditions, a lack of appreciation, and organisational support [11].

According to the guidelines of the World Health Organisation, a healthy work environment is characterised by ongoing collaboration between managers and workers to promote and maintain health in the workplace, ensure employee safety and well-being, and support the sustainability of the work environment [12]. 

In 2005, researchers from the University of Chile developed an instrument to measure the strategies that healthcare professionals use to cope with work-related stress. The Environmental Protectors Against Hospital Work Stress was created from qualitative research and initially included 45 items spread across 5 dimensions: organisation, workplace, leadership, physical environment, and teamwork/psychosocial environment. After content validation, three items were removed from the instrument. During the evaluation of the psychometric properties, two models were tested, containing 42 and 40 items, respectively, distributed across the five dimensions. The model showing the best fit in the confirmatory factor analysis was the one with 40 items, demonstrating acceptable reliability and validity [13]. 

Following cultural adaptation and content validation for Brazil [14], this study advances the validation process to assess the representativeness of ENPROS as a legitimate instrument for measuring the construct it aims to evaluate. Therefore, the aim of this study was to examine the psychometric properties of the Brazilian Portuguese version of the ENPROS scale.

## 2. Methods

### 2.1. Study Design, Setting, and Period

A methodological study, employing a quantitative approach, was conducted in two public hospitals in São Paulo state, Brazil, from January to October 2024. 

### 2.2. Study Population

The sample, selected for convenience, included physicians and nursing staff from all hospital sectors, excluding outpatient clinics, who had more than six months of work experience in these hospitals and had given their consent to participate. Individuals engaged in administrative activities, those on holiday, and those who did not report any conditions were excluded.

Although there is no gold standard for validating a new instrument, it is recommended that the sample size be at least four to ten times the number of items, with a minimum of 160 individuals to ensure adequate validity analysis [12,15]. Our analytical sample consisted of 431 participants.

### 2.3. Instruments

Two instruments were used for data collection. The Environmental Protectors Against Hospital Work Stress (ENPROS) scale was initially employed, an instrument developed in Chile in 2005 to evaluate environmental factors that serve as protectors against occupational stress among hospital staff. It was recently translated and culturally validated in Brazil [14]. ENPROS contains 40 items spread across 5 main dimensions: Organisation (7 items), Workplace (4 items), Leadership (8 items), Physical Environment (7 items), and Teamwork and Psychosocial Environment (14 items). The scale assesses the significance attributed to various aspects of the work environment on a 5-point scale, ranging from 1 (no importance) to 5 (extremely important). Higher scores reflect a more positive perception of protective environmental factors against occupational stress within the hospital setting. At the end of the instrument, there is a section for the sociodemographic data of the participants.

The second instrument was the Job Stress Scale, translated and adapted into Brazilian Portuguese by Alves et al. [16]. The scale consists of 11 items: 5 assessing psychological demands at work (items A, B, C, D, E) and 6 evaluating control over work (items F, G, H, I, J, K). Each item is scored on a five-point scale: (0) never, (1) hardly ever, (2) rarely, (3) sometimes, and (4) often. Additionally, the scale includes six items related to social support (items L, M, N, O, P, Q), each scored on a four-point scale: (1) entirely disagree, (2) disagree, (3) agree, and (4) fully agree. The scale yields a total score ranging from 0 to 20 for psychological demands, 0 to 24 for control, and 6 to 24 for social support. The scores are categorised as low or high: for psychological demands, low (score ≤ 12) and high (score > 13); for control, low (≤17) and high (>18); and for social support, low (≤21) and high (>22). The average response time for completing both instruments among the participants was approximately 20 min.

Forty-two participants from the original sample who were available and agreed to complete the instrument again were reassessed between 7 and 14 days after their first interview to evaluate temporal stability (test–retest analysis) [17,18].

### 2.4. Statistical Analysis

#### 2.4.1. Descriptive Analysis

The data were initially assessed for normality using the Shapiro–Wilk test, outliers with box plots, and missing data. For the scale items, no missing data were found; however, for age, four data points (0.9%) were missing, and for professional experience, twenty-one (4.9%) were missing. The number of missing data points was reported for each variable, and the analysis was performed excluding these cases. To describe the sample, the continuous variables were reported as the means and standard deviations, while the categorical variables were shown as percentages. 

#### 2.4.2. Exploratory Factor Analysis

The Kaiser–Meyer–Olkin (KMO) test and Bartlett’s sphericity test were used to evaluate the sample’s suitability for factor analysis. These statistical procedures help determine whether factor analysis is appropriate, indicating the quality of the sample and factorability of the data. The criteria for adequacy included a KMO value above 0.50 and a significant result in Bartlett’s sphericity test (*p* < 0.05) [19].

To test the hypothesis regarding the number of factors each scale adopts, Horn’s parallel analysis was used with the minimal residual factorisation method (MINRES), summarised by a scree plot. The traditional correlation matrix and Oblimin rotation were applied to identify the scales’ underlying structure. Factor loadings, commonality, the complexity of the original items, the cumulative variance, and the objective function (OF) were obtained through exploratory factor analysis (EFA).

Two criteria were used for retaining factors during the extraction stage: absolute factor loadings above 0.30 and at least three items per factor. 

#### 2.4.3. Confirmatory Factor Analysis

In the confirmatory factor analysis (CFA), various fit indices were employed to evaluate the model’s suitability to the observed data. The comparative fit index (CFI) and the Tucker–Lewis index (TLI) were considered, with values from 0.90 to 0.95 indicating an acceptable fit, while values of ≥0.95 denoted a good fit. The root mean square error of approximation (RMSEA) was interpreted as indicating good fit within the range of 0.05 to 0.08, with *p* < 0.05. The standardised root mean square residual (SRMR) was also examined, with acceptable values set at ≤0.08. Furthermore, the minimum discrepancy of confirmatory factor analysis (CMIN) and the CMIN/degrees of freedom ratio were calculated. Standardised loadings were considered satisfactory when exceeding 0.30 [20].

#### 2.4.4. Convergent Validity

In the absence of an equivalent instrument that could be considered the “gold standard” and meet the methodological excellence criteria required for this study, the Job Stress Scale was used as the parameter for analysing convergent validity. The correlation between the ENPROS and Job Stress Scale scores was calculated using Spearman’s correlation coefficient, interpreted as follows: <0.4 = weak correlation; 0.4 to 0.6 = moderate correlation; >0.6 = strong correlation [21].

#### 2.4.5. Reliability

Reliability analysis was conducted based on internal consistency and temporal stability, as assessed through test–retest analysis. The internal consistency of the scale and its dimensions were evaluated using Cronbach’s alpha coefficient, with values above 0.70 considered acceptable [22]. Temporal stability was analysed using the intraclass correlation coefficient (ICC), with values of <0.4, 0.4 to 0,6, 0.6 to 0.75, and >0.75 regarded as weak, fair, good, and excellent, respectively [18]. 

The data were analysed using the Statistical Package for the Social Sciences (SPSS) version 21.2 and R software version 2.3.28. The level of significance was set at 5% (*p* < 0.05) for all the statistical tests.

### 2.5. Ethical Aspects

This study was approved by the Research Ethics Committee of the Botucatu School of Medicine (Certificate No. 4.007.407). It was reported in accordance with the Strengthening the Reporting of Observational Studies in Epidemiology (STROBE) guidelines [23] and adhered to the Consensus-based Standards for the Selection of Health Measurement Instruments (COSMIN) [24]. It was conducted in line with the ethical principles outlined in the Declaration of Helsinki [25].

## 3. Results

The sample included 431 participants from public tertiary hospitals, the majority of whom were female (342; 79.4%). The mean age of the participants was 37.82 ± 9.76 years. The participants had an average professional experience of 11.30 ± 8.83 years, and their average length of employment at the current institution was 7.53 ± 6.57 years. Regarding marital status, married individuals (184; 42.7%) and single individuals (161; 37.3%) were most common. A total of 380 participants (88.2%) were involved in healthcare activities (Table 1). The nursing team was the largest group, comprising 127 (29.5%) nurses and 176 (40.8%) nursing technicians. Overall, 25.7% worked in intensive care units (ICUs), 15.5% in the emergency department, 28.8% in wards, 20.2% in surgical centres, and 31.5% in other sectors, with 13.7% reporting that they worked in more than one sector.

### 3.1. Construct Validity

#### 3.1.1. First Step—Confirmatory Factor Analysis (CFA)

Confirmatory factor analysis was performed on an initial model with five dimensions, using a robust error estimate. However, the fit indices of the final model did not meet acceptable standards: comparative fit index (CFI) at 0.791; robust Tucker–Lewis Index (TLI) at 0.777; RMSEA at 0.074; ^2^/df at 3.01 with *p* < 0.000. The first three values suggest a poor fit, as CFI < 0.90; TLI < 0.90; RMSEA > 0.06, while the last value indicates a good fit, since ^2^/df < 5. Therefore, we tested alternative models with fewer factors. 

Given the absence of previous models proposing two, three, or four factors, the one-factor model was used for comparison with the five-dimensional model. The one-factor model produced the following statistics: CFI = 0.657, TLI = 0.639, RMSEA = 0.100, and ^2^/df = 1.93 with *p* = 0.046. All the analytical options were also examined using modification indices and item exclusion.

#### 3.1.2. Second Step—Exploratory Factor Analysis (EFA)

**Figure 1:**
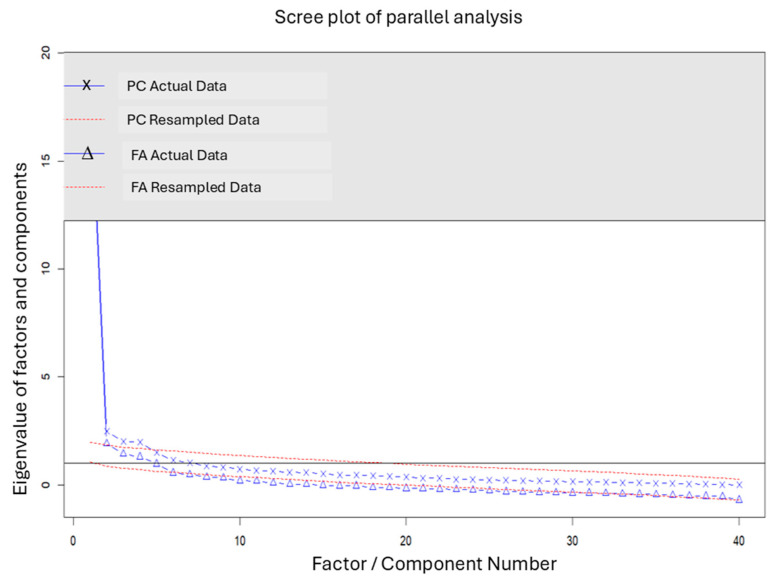
Screen plot of Horn’s parallel analysis with 40 ENPROS items.

The KMO measure was 0.93, and Bartlett’s test of sphericity showed *p* < 0.001, confirming the sample’s adequacy for EFA. Horn’s parallel analysis identified five oblique factors with eigenvalues above 1.0 (Figure 1).

EFA was conducted to examine how the items were grouped based on the five extracted factors (Table 2). Only one factor, which included items 20 and 21, was excluded despite having an acceptable total explained variance; however, a minimum of three items per factor is recommended to ensure it is well defined. The instrument explained 39.3% of the variance in the data. Communality values ranged from 0.26 (item 34) to 0.75 (item 21), indicating varying levels of explanation for the items within the factors. Table 2 displays the factor loadings, communality values, and complexity indices derived from this analysis.

Table 3 displays the percentage distribution of responses to the items categorised by dimension (Organisation, Post, Leadership, Environment, and Work), along with the corresponding item-total coefficients (ITC) and Cronbach’s alpha coefficients.

#### 3.1.3. Third Step—Confirmatory Factor Analysis

**Figure 2:**
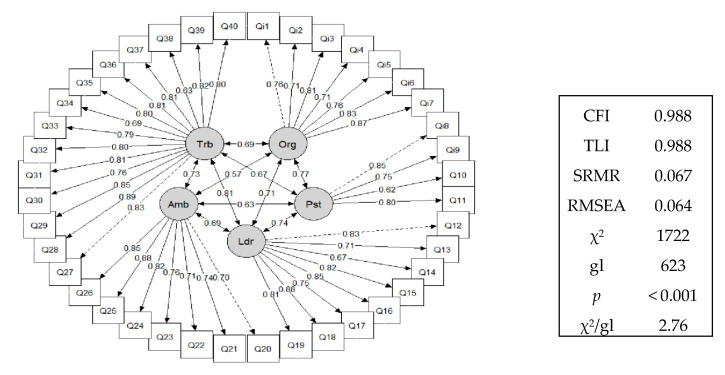
Path diagram of CFA for new dimensional structure of ENPROS.

After excluding items 20 and 21, the structure of the instrument was organised into four factors (F1, F2, F3, and F4), defined based on the analysis of the factorial groupings obtained in the preliminary stages, which confirms a redistribution of the items in contrast to the proposed model. The indices indicate the goodness-of-fit of the model to the data, as shown by the following: CFI = 0.988, TLI = 0.988, RMSEA = 0.064, SRMR = 0.067, χ2 = 1722, degrees of freedom (df) = 623, *p* < 0.001, with a χ2/df ratio = 2.76. These results empirically support the theoretical structure of the instrument, as demonstrated in Figure 2.

### 3.2. Concurrent Validity

Table 4 shows the correlations between the ENPROS domains and the Job Stress Scale, assessed using Spearman’s correlation coefficient test. Several correlations were statistically significant, especially with the social support (SUP) factor of the JSS, which had a negative correlation with D3 and D4, as well as between the D3 domain of the ENPROS and the psychological demand domain of the JSS.

### 3.3. Reliability

Reliability analysis was conducted based on the internal consistency of the instrument using Cronbach’s alpha coefficient and temporal stability (test–retest), with the intraclass correlation coefficient (ICC). The results indicated satisfactory levels of internal consistency for all ENPROS factors, with alpha values ranging from 0.92 (Factor 1), 0.85 (Factor 2), 0.75 (Factor 3), and 0.86 (Factor 4). The alpha value for the overall scale was 0.95. The Job Scale’s Cronbach’s alpha was 0.75.

Table 5 presents the means for the overall ENPROS score during the test–retest assessment of temporal stability. Little change was observed upon retesting, indicating acceptable levels of temporal stability close to the threshold for moderate reliability.

## 4. Discussion

The present study aimed to analyse the psychometric properties of the Brazilian Portuguese version of the Environmental Protectors Against Hospital Work Stress (ENPROS) scale. The sample included physicians, nurses, and nursing technicians. The nursing staff had an average tenure at the same hospital, indicating professional stability.

The distribution of professional categories in the sample showed a higher number of nursing technicians, followed by physicians and nurses (29.5%), reflecting the hierarchical structure and the increased demand for this professional group within the health team [26]. Considering the evidence about the influence of marital status on social support and, consequently, on stress levels among professionals, it was considered essential to incorporate sociodemographic variables into the analysis. This approach helps create a more robust methodological framework that accounts for the diverse situations of participants. Therefore, before conducting the factor analysis, the data’s suitability was assessed using the Kaiser–Meyer–Olkin (KMO) measure and Bartlett’s test of sphericity. The KMO value was 0.93, indicating a good fit for applying factor analysis, according to Kaiser’s criteria (1974) [27], which states that values above 0.80 are satisfactory. Bartlett’s sphericity test produced a statistically significant result (*p* < 0.001), showing that the correlations between items are significantly different from zero. These findings suggest that the data are suitable for identifying an underlying factorial structure, justifying the continuation of the factor analysis to evaluate the instrument’s construct validity.

The results showed strong internal consistency of the ENPROS, especially in the leadership, work, and organisation dimensions, which had high Cronbach’s alpha coefficients (>0.85). However, this study identified a ceiling effect, which occurs when scoring is uneven and characterised by the proportion of answers at the highest levels of the measurement [28]. Ceiling and floor effects can affect the sensitivity of the measure in question.

In the first stage, confirmatory factor analysis based on the original 5-dimensional 40-item model showed that the CFI (0.791) and TLI (0.777) values were below the recommended minimum of 0.90, indicating that the proposed model did not fit the observed data adequately. Although the RMSEA (0.074) was within the acceptable range, the significant chi-square value (*p* < 0.001) and other indices suggested that the model required adjustment, emphasising the need to conduct EFA. 

EFA was performed to develop a reasonable and comprehensive model that would produce a solid theoretical conceptual framework [29], based on the criterion of retaining at least three items per factor. Only one factor, with items 20 and 21, was excluded despite having an acceptable total explained variance; a minimum of three items per factor is recommended to ensure that the factor remains well defined and stable across samples [30]. As a result, the revised distribution of items within each dimension was as follows: D1 (Items 25 to 39), D2 (Items 1, 2, 3, 4, 5, 6, 7, 8, and 11), D3 (Items 10, 22, 23, 24, and 40), and D4 (Items 9, 12, 13, 14, 15, 16, 17,18, and 19).

Confirmatory factor analysis was subsequently performed after the instrument was reorganised into four factors. All the quality criteria for the goodness of fit of the model were satisfactory, with the final model demonstrating high standards of quality (CFI = 0.988, TLI = 0.988, RMSEA = 0.064, SRMR = 0.067, χ2 = 1722, df = 623, and *p* < 0.001, with a χ2/df ratio of 2.76, consistent with the literature). Furthermore, the results support the construct validity of the instrument, indicating that the items are appropriately organised into four theoretical factors, thereby allowing a comprehensive assessment of the factors associated with occupational stress.

The scale’s reliability was evaluated by examining its internal consistency using Cronbach’s alpha and its temporal stability through a test–retest analysis using the intraclass correlation coefficient (ICC). Cronbach’s alpha is a key method for assessing the reliability of instruments with multiple items [31]. Reliability was found to be excellent for the overall scale (α = 0.95), with factor loadings of 0.92 for Factor 1, 0.85 for Factor 2, 0.75 for Factor 3, and 0.86 for Factor 4. All the factors reached or exceeded the minimum acceptable threshold of 0.70, which is commonly suggested for instruments in development or initial validation [32]. Cronbach’s alpha correlations are strongly affected by the number of items in a measure [32]. 

Temporal stability was assessed using the Intraclass Correlation Coefficient (ICC), as recommended by Cosmin [33], which measures the level of agreement between measurements taken in both applications, typically spanning 7 to 14 days. This method is widely endorsed because it helps determine whether the instrument yields consistent results over time [34]. The sample of 42 participants was sufficiently large for ICC estimates between 0.40 and 0.60, according to Bujang and Baharum (2017) [35]. There was minimal discrepancy between the scores in the test and retest assessments, indicating satisfactory temporal stability and moderate reliability. Only Dimension 4 showed a low ICC (0.29), indicating that caution should be exercised when interpreting this dimension [35].

The assessment of convergent validity was carried out using Spearman’s correlation coefficient to examine the relationships between the ENPROS domains and the factors of the Job Stress Scale (JSS). Notably, the negative correlation observed between the D3 and D4 domains and the JSS social support factor (SUP) highlights that higher perceived social support is linked to lower perceptions of work environment stressors. Additionally, a significant correlation was identified between the D3 domain of ease and the Psychological Demand factor of the JSS, indicating that perceptions of psychological demands at work may be connected to previous negative experiences in leadership contexts.

The negative, statistically significant correlations between the ENPROS D3 (working conditions) and D4 (interpersonal relationships) domains and the SUP (social support) factor of the JSS (ρ = −0.13; *p* = 0.007 and ρ = −0.10; *p* = 0.048, respectively) suggest that lower levels of social support at work are linked to a perception of poorer conditions and relationships within the professional environment. Additionally, a positive correlation was found between D3 and psychological demands (ρ = 0.11; *p* = 0.026), indicating a connection between excessive workload and unfavourable aspects of working conditions. The other correlations were weak and non-significant, which may imply some independence between the assessed constructs or limited sensitivity of the measures in capturing these associations across certain dimensions.

The reliable and valid instrument can help foster a safe and healthy workplace that prioritises the preservation and enhancement of physical and mental health, providing a new perspective for healthcare providers working in hospital settings. This can empower workers, organisations, and society as a whole to achieve optimal well-being [36,37].

## 5. Limitations

This study acknowledges several limitations that should be considered. The sample consisted of healthcare professionals from only two hospitals, which limits the generalisability of the findings to other institutional contexts. Additionally, the use of a non-probabilistic sample, predominantly composed of women, presents another significant limitation regarding the representativeness of the results.

## 6. Conclusions

The results of this study show that the Brazilian Portuguese version of ENPROS has a four-dimensional structure with 38 items redistributed across these factors, demonstrating good reliability and a strong fit to the proposed factor structure. A notable strength is the availability of an innovative instrument for Brazil, which aligns with institutional guidelines aimed at proposing interventions to cope with work-related stress.

## Figures and Tables

**Table 1:** Sociodemographic and occupational characterisation of 431 participants of the study. Botucatu, SP, Brazil, 2025.

Variables	n	%
**Occupation**		
Physician	128	29.7
Nurse	127	29.5
Nursing technician	176	40.8
**Age**		
Missing data	4	0.9
Mean ± SD *	37.82 ± 9.76	
**Sex**		
Male	89	20.6
Female	342	79.4
**Activity**		
Administration	17	3.9
Care	380	88.2
Both	34	7.9
**Marital status**		
Single	161	37.3
Married	184	42.7
Separated/divorced	37	8.6
Widowed	6	1.4
Stable union	36	8.4
Other	7	1.6
**Work location**		
ICU	111	25.7
Emergency	67	15.5
Wards	124	28.8
Surgical centre	87	20.2
Other	136	31.5
More than one unit	59	13.7
**Professional experience**		
Missing data	21	4.9
Mean ± SD *	11.30 ± 8.83	
**Experience at institution**		
Mean ± SD *	7.53 ± 6.57	

* Mean (standard deviation).

**Table 2:** Factor loadings, communality (h2), and complexity (com) of the EFA. Botucatu, SP, Brazil, 2025.

	MR1	MR2	MR3	MR4	MR5	h2	com
Item 1	0.11	0.58	0.06	−0.02	−0.01	0.42	1.1
Item 2	−0.11	0.71	0.08	0.04	−0.02	0.48	1.1
Item 3	−0.01	0.71	0	−0.05	0.06	0.55	1.0
Item 4	−0.12	0.68	−0.17	0.19	0.04	0.48	1.4
Item 5	0.12	0.58	0.05	−0.07	0	0.43	1.1
Item 6	0.2	0.64	−0.02	−0.09	−0.03	0.51	1.2
Item 7	0.13	0.69	0	−0.05	0.01	0.58	1.1
Item 8	−0.12	0.48	0.14	0.09	0.23	0.44	1.9
Item 9	−0.2	0.26	0.13	0.14	0.39	0.34	2.9
Item 10	0.08	0.18	−0.23	0.34	0.13	0.31	2.9
Item 11	0.06	0.43	0.21	−0.12	0.15	0.42	2.0
Item 12	0.18	0.06	0.02	0	0.53	0.49	1.3
Item 13	−0.05	0.03	0	0.01	0.62	0.38	1.0
Item 14	−0.06	0.08	−0.01	−0.09	0.57	0.33	1.1
Item 15	−0.05	0.12	0.05	0.06	0.6	0.47	1.1
Item 16	0.04	−0.03	−0.07	0.21	0.68	0.56	1.2
Item 17	0.16	−0.01	0.03	−0.16	0.61	0.48	1.3
Item 18	0.22	0.12	0.07	−0.11	0.51	0.57	1.6
Item 19	0.1	0.06	0.06	0.04	0.54	0.45	1.1
Item 20	−0.02	−0.05	0.83	−0.03	0.04	0.68	1.0
Item 21	0.01	0.04	0.87	0.08	−0.08	0.75	1.0
Item 22	0.01	0.07	0.3	0.5	0.06	0.43	1.7
Item 23	0.04	0.01	0.26	0.51	0.23	0.52	2.0
Item 24	0.17	0.02	0.05	0.43	0.28	0.46	2.1
Item 25	0.38	0.01	0.34	0.07	0.16	0.51	2.4
Item 26	0.36	0.11	0.2	0.13	0.14	0.43	2.5
Item 27	0.42	0.1	0.03	0.21	0.22	0.52	2.2
Item 28	0.54	0.08	0.08	0.04	0.23	0.62	1.5
Item 29	0.53	0.09	−0.02	−0.05	0.28	0.59	1.6
Item 30	0.6	−0.03	0.08	−0.27	0.21	0.6	1.7
Item 31	0.69	0.1	0.1	−0.29	0.04	0.68	1.4
Item 32	0.54	0.24	0.02	0.01	0.02	0.5	1.4
Item 33	0.35	0.15	0.05	0.1	0.21	0.41	2.3
Item 34	0.34	0.14	−0.05	0.12	0.1	0.26	1.9
Item 35	0.63	0.05	−0.05	0.26	0.06	0.56	1.4
Item 36	0.61	0.04	−0.06	0.27	0.07	0.54	1.4
Item 37	0.75	0	0.08	0.03	0.01	0.62	1.0
Item 38	0.5	0.05	0.18	−0.02	−0.04	0.34	1.3
Item 39	0.5	0.14	0.02	0.37	0.01	0.54	2.0
Item 40	0.36	0.17	−0.05	0.41	0.11	0.52	2.5
Eigenvalue	4.68	3.80	2.09	1.65	3.48		
%EV	11.7	9.5	5.2	4.1	4.1		
%EV accumulated	11.7	21.2	26.4	39	39.3		

**Table 3:** Percentage distribution of ENPROS scores and reliability indicators (ITC and CA) based on 431 completed questionnaires.

Item	1	2	3	4	5	ITC	CA s/o Item ^1^	CA
Organization								
O01	0.2	1.4	8.1	36.4	53.8	0.576	0.845	
O02	0.7	2.6	6.7	25.1	65.0	0.605	0.841	0.858
O03	0.5	1.6	8.8	31.8	57.3	0.675	0.831	
O04	0.2	0.9	3.0	16.7	79.1	0.569	0.847	
O05	0.9	1.9	13.5	30.4	53.4	0.594	0.844	
O06	0.0	1.6	9.7	33.6	55.0	0.677	0.831	
O07	0.2	0.9	7.4	30.9	60.6	0.701	0.828	
Post								
P08	0.2	0.2	3.2	26.0	70.3	0.552	0.589	0.682
P09	0.0	0.9	2.6	18.1	78.4	0.438	0.647	
P10	3.2	5.8	18.3	33.9	38.7	0.419	0.704	
P11	0.7	1.9	10.4	32.5	54.5	0.582	0.534	
Leadership								
L12	0.2	0.7	5.1	24.1	69.8	0.642	0.840	
L13	0.5	0.9	7.4	34.1	57.1	0.598	0.845	0.861
L14	0.2	1.9	7.7	31.6	58.7	0.531	0.854	
L15	0.2	0.7	4.2	23.7	71.2	0.613	0.843	
L16	0.2	0.0	2.3	13.9	83.5	0.611	0.846	
L17	0.2	1.2	6.7	29.0	62.9	0.609	0.844	
L18	0.5	1.6	4.4	29.0	64.5	0.681	0.835	
L19	0.2	1.4	4.4	21.6	72.4	0.613	0.843	
Environment								
E20	2.1	7.0	20.6	28.8	41.5	0.561	0.784	
E21	1.2	6.0	17.6	30.2	45.0	0.642	0.762	0.806
E22	0.2	0.5	3.5	22.0	73.8	0.501	0.790	
E23	0.2	0.7	4.2	22.3	72.6	0.546	0.783	
E24	0.0	0.5	2.6	23.0	74.0	0.456	0.797	
E25	0.7	1.6	12.3	29.2	56.1	0.630	0.764	
E26	0.7	1.9	11.6	28.8	57.1	0.540	0.781	
Work								
W27	0.2	0.5	4.6	29.0	65.7	0.653	0.909	
W28	0.2	0.2	5.6	31.8	62.2	0.743	0.907	0.916
W29	0.2	0.9	5.3	34.6	58.9	0.720	0.907	
W30	0.7	2.1	14.4	35.3	47.6	0.636	0.910	
W31	0.5	2.8	14.2	41.5	41.1	0.690	0.908	
W32	0.5	1.2	5.3	38.3	54.8	0.684	0.908	
W33	0.7	0.5	3.2	31.1	64.5	0.604	0.911	
W34	2.1	0.2	4.2	23.4	70.1	0.481	0.916	
W35	0.2	0.5	6.0	28.5	64.7	0.687	0.908	
W36	0.2	0.5	4.9	31.1	63.3	0.666	0.909	
W37	0.9	1.2	11.1	31.6	55.2	0.732	0.906	
W38	2.1	6.7	16.5	27.1	47.6	0.524	0.918	
W39	0.2	0.7	5.1	19.7	74.2	0.638	0.910	
W40	0.2	0.0	2.3	20.4	77.0	0.587	0.912	

ITC: item-total correlation; CA: Cronbach’s alpha; ^1^ CA for 40 items was 0.949.

**Table 4:** Spearman’s correlation coefficients between the dimension scores of the ENPROS and the Job Stress Scale. Botucatu, SP, Brazil, 2025.

	JOB		PSYCH		CTRL		SUP	
	Rho	*p*	Rho	*p*	Rho	*p*	Rho	*p*
ENPROS	0.03	0.604	0.04	0.394	0.04	0.390	−0.09	0.073
D1	0.01	0.783	0.01	0.803	0.01	0.842	−0.06	0.220
D2	0.04	0.509	0.05	0.285	0.08	0.102	−0.09	0.061
D3	0.02	0.762	0.11	0.026	0.01	0.879	−0.13	0.007
D4	0.01	0.953	0.05	0.327	0.03	0.517	−0.10	0.048

JOB (Job Stress Scale); PSYCH (psychological demand); CTRL (control over work); SUP (social support); Rho: rho de Sperman.

**Table 5:** Distribution of temporal stability (test–retest) of ENPROS scale and subscales (n = 42).

Variable	Test	Retest	ICC (95%CI)	*p*
ENPROS38	170 (157–178)	173 (162–182)	0.45 (0.18–0.66)	0.001
D1	66.5 (60–73)	66.5 (61.3–72.8)	0.59 (0.35–0.76)	<0.001
D2	39.5 (36–42)	40.5 (37.3–43.8)	0.40 (0.11–0.62)	0.004
D3	23 (21–24)	23 (21–24)	0.46 (0.19–0.67)	0.001
D4	41.5 (38.3–44.8)	42 (39–45)	0.29 (−0.01–0.54)	0.0030

## Data Availability

The datasets generated and/or analysed during the current study are not publicly available to protect the anonymity of the respondents, but they can be obtained from the corresponding author upon reasonable request.
